# Biodegradation of the Alkaline Cellulose Degradation Products Generated during Radioactive Waste Disposal

**DOI:** 10.1371/journal.pone.0107433

**Published:** 2014-09-30

**Authors:** Simon P. Rout, Jessica Radford, Andrew P. Laws, Francis Sweeney, Ahmed Elmekawy, Lisa J. Gillie, Paul N. Humphreys

**Affiliations:** Department of Chemical and Biological Sciences, School of Applied Sciences, University of Huddersfield, Huddersfield, United Kingdom; Belgian Nuclear Research Centre SCK•CEN, Belgium

## Abstract

The anoxic, alkaline hydrolysis of cellulosic materials generates a range of cellulose degradation products (CDP) including α and β forms of isosaccharinic acid (ISA) and is expected to occur in radioactive waste disposal sites receiving intermediate level radioactive wastes. The generation of ISA's is of particular relevance to the disposal of these wastes since they are able to form complexes with radioelements such as Pu enhancing their migration. This study demonstrates that microbial communities present in near-surface anoxic sediments are able to degrade CDP including both forms of ISA via iron reduction, sulphate reduction and methanogenesis, without any prior exposure to these substrates. No significant difference (n = 6, p = 0.118) in α and β ISA degradation rates were seen under either iron reducing, sulphate reducing or methanogenic conditions, giving an overall mean degradation rate of 4.7×10^−2^ hr^−1^ (SE±2.9×10^−3^). These results suggest that a radioactive waste disposal site is likely to be colonised by organisms able to degrade CDP and associated ISA's during the construction and operational phase of the facility.

## Introduction

The current strategy for the management of the UK's radioactive waste is a single Geological Disposal Facility (GDF) providing suitable, safe containment of the national waste inventory. One illustrative concept for the disposal of long lived, Intermediate Level Wastes (ILW) and some Low Level Wastes (LLW) is that of a cementitious backfilled facility which will re-saturate post closure [1]. Although the facility in general will be backfilled with a cementitious grout, not all the waste will be encapsulated with cement allowing lower pH environments to be present within the waste. Such a facility is expected to develop anoxic conditions soon after closure due to the removal of oxygen by the corrosion of the steel waste containers. This will result in an anoxic, alkaline environment which in combination with the host rock, will provide a multi barrier system for the containment of the radionuclide inventory [1].

By 2010, the U.K. held an estimated 2,000 tonnes of cellulosic ILW composed of packaging materials, disposable clothing and surface wipes and a further 100,000 tonnes of cellulosic LLW [2]. The chemical hydrolysis of cellulose under anoxic, alkaline conditions is a well described process [3] in which amorphous cellulose is degraded via the peeling reaction to the α and β forms of isosaccharinic acid (ISA) and a range of organic acids including formic, glycolic and acetic [4]. Of these water soluble products, ISA has received considerable attention on account of its ability to form complexes with radionuclides present in the wastes [5]–[7]. The construction and operational phases of a GDF provide an opportunity for the microbial contamination and colonisation of the facility by microorganisms from the near-surface environment [8]. The microbial degradation of ISA may have a significant impact on the evolution of GDF and the migration of the radioelements present.

Within the wider environment, ISA is generally absent, although it is present in the black liquor resulting from the Kraft paper pulping process [9]. Studies of industrially contaminated sites suggest that ISA degrading microbial populations may evolve within decades [10]–[12]. Other studies have shown that ISA is capable of being degraded under aerobic and denitrifying conditions, conditions unlikely to be seen in the near field of a GDF [9], [11]. The presence of significant amounts of steel (construction materials and waste packages) within a GDF mean that corrosion processes will promote the generation of reducing conditions and generate ferric iron phases that may support microbial iron reduction [1]. In addition, groundwater ingress is likely to provide sulphates that will be able to support microbial sulphate reduction [13]. Consequently, both iron reduction and sulphate reduction along with methanogenic processes may play an important role in the development of the ambient geochemistry within a GDF [8]. ISA and other organic compounds generated by the chemical hydrolysis of cellulose are potential substrates for these microbial processes.

The aim of this study was to determine the ability of the microbial communities in anoxic near surface sediments to degrade CDP and associated ISA under iron reducing, sulphate reducing and methanogenic conditions. As such, this study investigates the microbial processes that may take place at the interface between ungrouted cellulosic wastes and the cementitious backfill and at the interface between the repository and the host rock that is receiving an ISA containing plume.

## Experimental Procedures

### Sediment samples

Sediment samples were taken from the Leeds/Liverpool canal at the University of Huddersfield (ordnance co-ordinates, SE 14890 16416); further samples were taken from reed beds at the National Coal Mining museum Wakefield (SE 25076 16368). Samples were taken using a weighted sampler and stored under anoxic conditions in sealed plastic containers at room temperature; samples were transferred into microcosms within 14 days of collection. The permissions of the University of Huddersfield and National Coal Mining Museum were acquired prior to sampling.

### Production of cellulose degradation products (CDP)

CDP was prepared in a similar fashion to that of Cowper et al [21] and standard laboratory tissue (Pristine paper hygiene, London, UK) was used as a cellulose source for degradation. Laboratory tissue (200 g) was added to 1.8 l of N_2_ flushed 0.1 M NaOH and 10 g l^−1^ Ca(OH)_2_ in a pressure vessel. The pressure vessel was sealed and made anoxic by flushing the headspace with nitrogen for 30 minutes and then placed in an incubator at 80°C for 30 days. After 30 days, the vessel was allowed to cool before the resultant liquor was sterile filtered under a nitrogen atmosphere. Bottles of CDP were covered with foil to exclude light and stored under a nitrogen atmosphere. The composition of the synthesised CDP can be found in supplementary table (Table S1 in File S1).

### Microcosm set up

Sediment samples were diluted 2-fold in the anaerobic mineral media specified in BS14853 [22] to a volume of 450 ml in 500 ml reaction vessels fitted with inlet and outlet ports to allow for the addition and removal of samples and a third port fitted with a septum to allow headspace gas sampling. Following an initial feed of 50 ml of CDP, microcosms were batch fed under nitrogen on a weekly cycle by replacing 50 ml of the microcosm contents with 50 ml of fresh CDP followed by a further 20 minutes of nitrogen flushing. This feeding cycle was continued for 24 weeks prior to sampling. Iron and sulphate reducing conditions were established through the additions of excess quantities of calcined iron (III) oxide (Fisher Scientific Ltd, UK) and sodium sulphate (Fisher Scientific Ltd, UK) relative to the moles of organic carbon present in the CDP. Methanogenic conditions were established through the absence of iron (III) or sulphate. The iron (III) oxide employed was identified as haematite via X-ray diffraction analysis (Bruker D2 phaser and diffraction patterns recorded using Cu-K_α_ radiation (*λ* = 1.54184 Å) utilising a LYNXEYE detector) and comparison with Bragg peaks obtained from the Powder Diffraction File database (Figure S1 in File S1). Previous authors have noted that sorption reactions do not occur between ISA and other CDP components and haematite [21], supporting the selection of this source of iron (III) for these microcosm experiments. Surface areas and pore sizes were determined by nitrogen adsorption at 77K using an ASAP2020 (Micromeritics Instrument Corp).

In addition, three control microcosms amended with 50 µg ml^−1^ chloramphenicol were set up containing the same proportions of mineral media, sediment and CDP and sampled on a daily basis.

### Chemical analysis

In brief, 5 mL samples were taken on a daily basis over 3 consecutive feed cycles under a nitrogen atmosphere. Samples were centrifuged at 9000×*g* for 10 minutes, the resulting supernatant was then sterile filtered using a 0.45 µm filter and kept at 4°C prior to use, subsequent analysis of samples was carried out under ambient conditions. In addition, 0.9 ml of sample was acidified with 0.1 ml of phosphoric acid and frozen at −20°C for volatile fatty acid analysis. The presence and concentration of volatile fatty acids was determined using a gas chromatograph (HP GC6890, Hewlett Packard, UK) fitted with a HP-FFAP column (Agilent Technologies) and a flame ionization detector under the following conditions: an initial temperature of 95°C for 2 minutes, followed by an increase to 140°C at a ramp rate of 10°C min^−1^ with no hold, followed by a second ramp to 200°C at a ramp rate of 40°C min^−1^ with a hold of 10 minutes, falling to a post run temperature of 50°C. Total organic carbon analysis was carried out using a Shimadzu TOC 5050A. ISA concentrations in both the alpha and beta conformations were measured using high performance anion exchange chromatography and pulsed amperometric detection on a Dionex 3000 Ion chromatography system (Dionex, Camberly, UK) employing a Dionex Carbopac PA20 column (3×150 mm, 6.5 µm particle size) and eluting with aqueous sodium hydroxide (0.05 mol l^−1^) against a range of standards [23]. The volume of gas produced was measured using a Quick Scan 1.8c apparatus (Challenge Technology, Arkansas, US), the headspace gas composition was determined using an Agilent 6850 gas chromatograph with a thermal conductivity detector and GS-Q column operating at a column temperature of 30°C and a detector temperature of 200°C. The soluble iron concentration was measured spectrophotometrically using a ferrozine extraction method described previously [24]. The sulphate concentration was measured via ion chromatography using amperometric detection on a Metrohm 850 Professional IC (Metrohm, Cheshire, UK) employing a Metrohm Metrosep A Supp 5 column (4×150 mm, 5 µm particle size) eluted with sodium carbonate and sodium hydrogen carbonate (3.2 mmol l^−1^, 1.0 mmol l^−1^ respectively) alongside a range of standards. The sulphide concentration was measured using a micro ION electrode LIS146AGSCM (Lazar Labs, US) calibrated against a range of standards. SEM analysis was carried out using an FEI Quanta FEG 250 equipped with energy-dispersive x-ray spectroscopy.

### DNA extraction and direct/nested PCR

A 50 ml sample was removed from each microcosm and centrifuged at 9000×*g* for 10 minutes at 4°C, 5 ml of supernatant was retained and mixed with the pellet to give a concentrated suspension. Microbial DNA was extracted from each sample using a PowerSoil DNA isolation kit (MoBio laboratories, California, US). Extracted DNA was diluted to a concentration of approximately 100 ng/µl and PCR carried out using a range of primers (Table S2 in File S1) in accordance with previously published methods [25]. When PCR product was not observed through direct PCR, the relevant 16S rDNA amplification product was used to perform a nested reaction using the associated primers. In addition, a range of control DNA samples (Table S3 in File S1) were used to validate the results of each PCR step.

#### Statistical Analysis

All statistical analysis was carried out using IBM SPSS V 20 for Windows, data were checked for their normality of distribution and equality of variance prior to ANOVA.

## Results and Discussion

**Figure 1:**
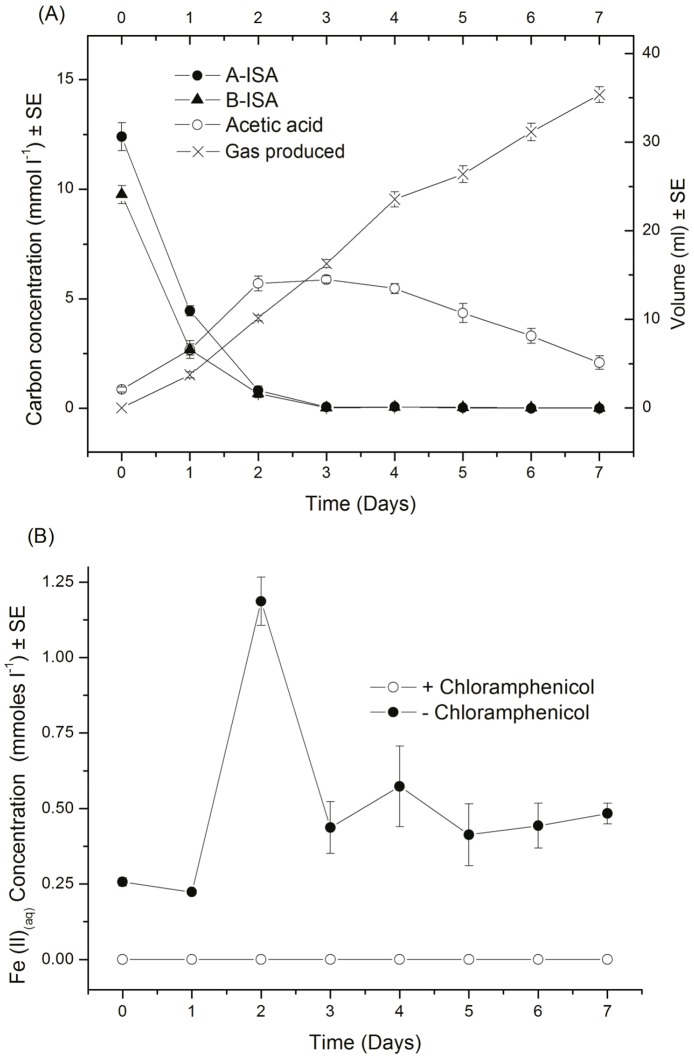
Organic chemical evolution of iron reducing reactors (A) and iron (II) (aq) evolution versus chloramphenicol treated control (B) (n = 6).

**Figure 2:**
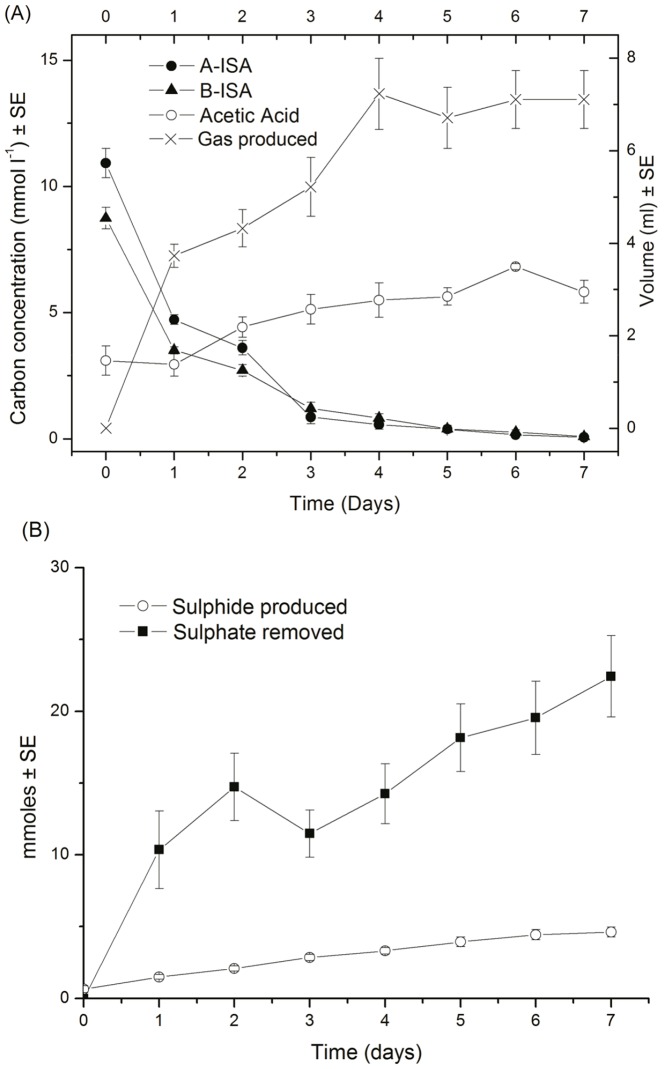
Organic chemical evolution of sulphate reducing reactors (A) and sulphate removal and sulphide production (B) (n = 6).

**Figure 3:**
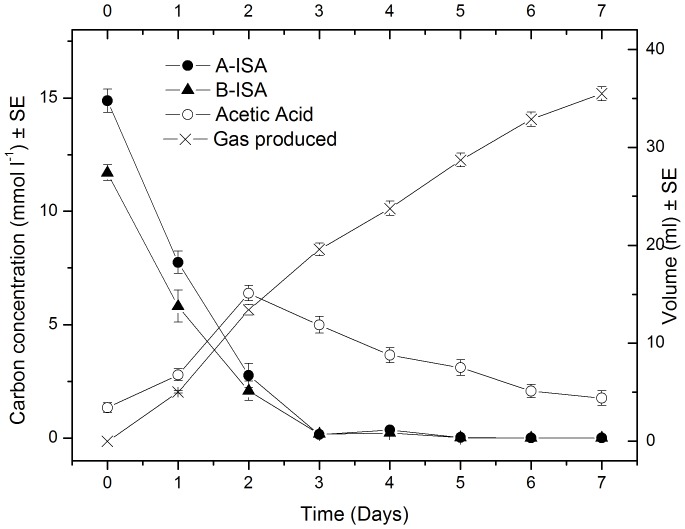
Chemical evolution of methanogenic reactors (n = 6).

Across all microcosms, under iron reducing, sulphate reducing and methanogenic conditions, a significant proportion of organic carbon removal was associated with α and β ISA metabolism with no apparent difference between α and β ISA consumption profiles (Figures 1A, 2A and 3). Fermentation processes were evident by the generation of acetic acid, which was the most prevalent volatile fatty acid formed, although other longer chain fatty acids including propionic, isobutyric, butyric and isovaleric acids were produced in sub mM concentrations (Figure S2 in File S1).

**Figure 4:**
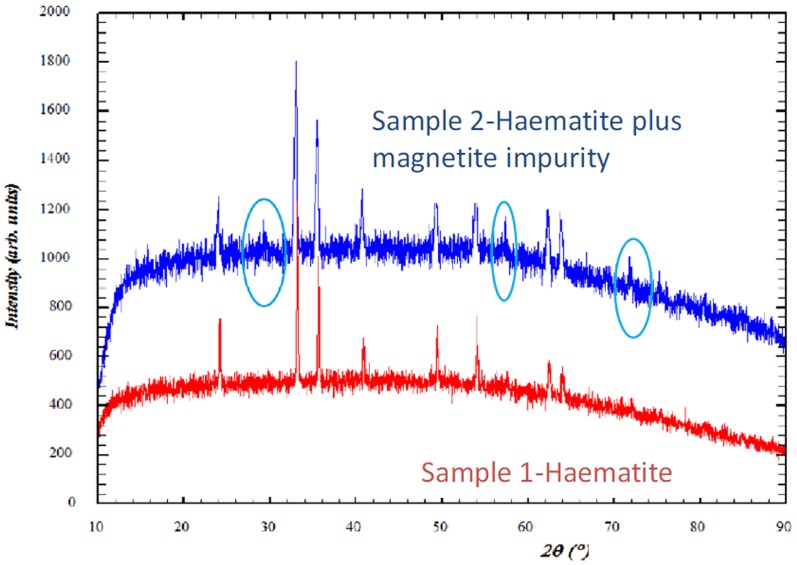
XRD patterns for the iron oxide used in microcosms (Sample 1), identified as haematite through comparison with diffraction database (peaks at 24, 33, 36, 41, 49 54, 62 and 64 2 Theta) and pattern at the end of the sampling period (Sample 2), where haematite contained an impurity, determined as magnetite through comparison with diffraction database (peaks at 30, 58 and 74 2 Theta, circled).

**Figure 5:**
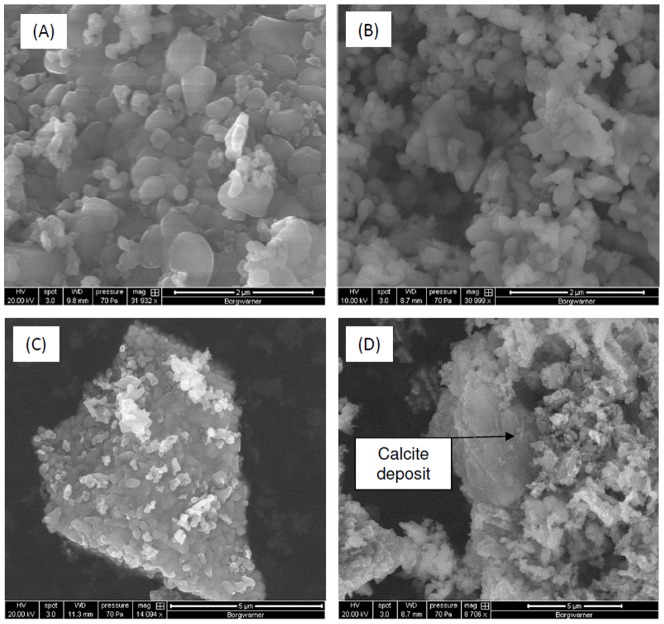
Scanning electron micrographs of virgin calcined iron (III) oxide (A, C) and iron (III) oxide following incubation under iron reducing conditions (B, D).

In microcosms amended with haematite, iron reduction was indicated by the generation of Fe (II)(B) which coincided with the removal of both forms of ISA (Figure 1A). This contrasts with the associated control reactors where no Fe (II) generation or ISA removal was observed. In these iron reducing systems the fermentation of at least a portion of the ISA was illustrated by the initial generation of acetic acid. However, by the end of the incubation period, acetic acid levels had significantly reduced (p<0.05) indicating its subsequent degradation (Figure 1A, B). The Fe (II) profiles indicate an initial generation followed by a reduction to a lower resting level. This profile is consistent with the precipitation of Fe (II) containing mineral phases, with the final solution phase concentrations determined by precipitation/dissolution reactions. This profile and the resting Fe (II) concentrations are also consistent with previously published data on haematite driven iron reduction systems [14]. XRD analysis confirmed the generation of Fe (II) mineral phases, in particular magnetite, that were absent from the original haematite (Figure 4). The presence of biogenic magnetite in bulk Fe (III) oxides has also been observed by previous authors employing XRD [15]. The surface area (from 4.4 m^2^ g^−1^ to 13.8 m^2^ g^−1^) and associated porosity (0.02 cm^3^/g to 0.04 cm^3^/g) of the haematite increased following iron reduction. This increased porosity was confirmed by SEM (Figure 5 A, B) which in conjunction with energy-dispersive x-ray spectroscopy (Figure S3 in File S1) confirmed the formation of calcite on the haematite surface (Figure 5 C, D). This suggests that calcite formation is occurring due to biogenic CO_2_ reacting with calcium present in the CDP. In contrast, both magnetite and calcite were absent from the sediment remaining in the control microcosms.

The formation of methane indicated that methanogens were also active alongside fermentative and iron reducing communities, suggesting that the crystalline nature of the Fe (III) source facilitates the presence of methanogenesis by limiting the rate of iron reduction. Haematite is known to support a lower rate of iron reduction than more amorphous Fe (III) phases or complexed Fe (III) [16]. Stoichiometric calculations [17] indicated that methanogenesis and accumulated acetic acid accounted for only 18% of the degraded ISA, confirming the role of iron reduction as the primary metabolic process within the system.

Of the Clostridia clusters investigated, direct and nested PCR approaches indicated that cluster IV was more abundant than clusters III and XIV, with cluster I being undetectable (Table 1). Iron reduction may be attributed to a mixture of *Geobacter* sp and organisms from sulphate reducing bacteria (SRB) groups 1, 3, 4, and 5. Previous authors have noted the ability of SRBs to enzymatically reduce Fe (III) from these groups [18]. Methanogenic bacteria capable of acetoclastic and hydrogenotophic metabolism were also present within this community (Table 1).

**Table 1:** DNA analysis by direct and nested PCR techniques.

		Terminal Electron Acceptor					
		Iron		Sulphate		Carbon Dioxide	
Species	Size	D	N	D	N	D	N
Clostridium I	820	-	-	-	-	-	-
Clostridium III	720	-	+	+	+	+	+
Clostridium IV	580	+	+	+	+	-	-
Clostridium XIV	620	-	+	+	+	+	+
Methanococcales	340	-	+	-	-	-	+
Methanobacteriales	340	+	+	-	-	+	+
Methanomicrobiales	550	+	+	-	-	+	+
Methanosarcinales	350	+	+	-	-	+	+
Methanosaeta	250	+	+	-	-	+	+
SRB group 1	702	-	+	+	+	-	-
SRB group 2	1120	-	-	-	-	-	-
SRB group 3	840	+	+	+	+	-	-
SRB group 4	1150	-	-	+	+	-	-
SRB group 5	860	+	+	+	+	-	-
SRB group 6	620	+	+	+	+	-	-
Geobacter	300	+	+	+	+	N	N
Shewanella	1040	-	-	-	-	N	N

N-not sampled.

**Figure 6:**
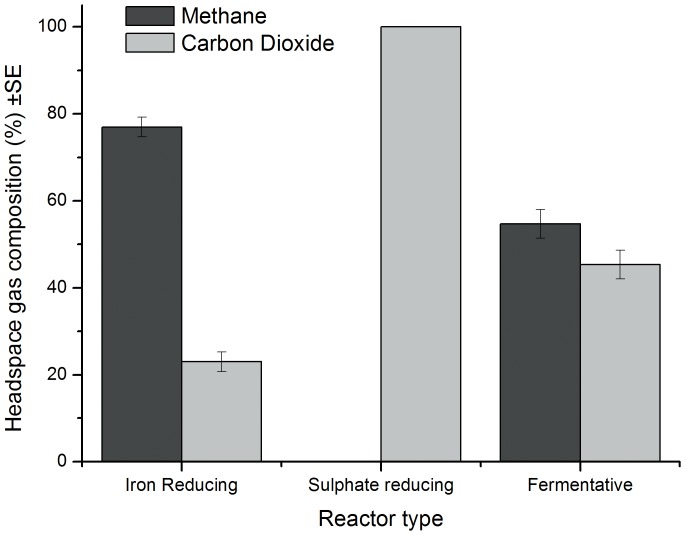
Composition of microcosm headspace gases (n = 6).

Unlike iron reduction, the presence of sulphate allowed SRBs to dominate the terminal electron accepting processes as indicated by the absence of evolved methane within the headspace of these microcosms (Figure 6). Sulphide was generated in the aqueous phase as sulphate was removed (Figure 2B); no free sulphide was detected in the associated control microcosms. The accumulation of acetic acid up to day 6 suggests that sulphate reduction of acetic acid is occurring at a slower rate than its generation. Through direct PCR the presence of groups 1, 3, 4, 5 and 6 sulphate reducing bacteria as described by [19] were observed alongside groups III, IV and XIV of the Clostridia.

In methanogenic microcosms (Figure 3) the removal of both forms of ISA was associated with the production and removal of acetic acid and the generation of methane which comprised 54.7%±3.3 of the gas generated. Direct and nested PCR confirmed the presence of Clostridia groups III and XIV and all five methanogen groups investigated.

Degradation rates for ISA under anoxic conditions are not available in the literature, consequently first order degradation rates were calculated from the iron reducing, sulphate reducing and methanogenic α and β ISA removal data. No significant difference (ANOVA, n = 6, p = 0.118) was found between the degradation rates of either α and β ISA under iron reduction, sulphate reduction or methanogenic conditions, giving an overall ISA degradation rate of 4.7×10^−2^ hr^−1^ (n = 36, SE±2.9×10^−3^). These data support a two stage degradation model for ISA with fermentation to short chain volatile fatty acids being the dominant, rate limiting step across all three consortia, followed by the iron reduction, sulphate reduction and methanogenesis of the fermentation end products.

**Figure 7:**
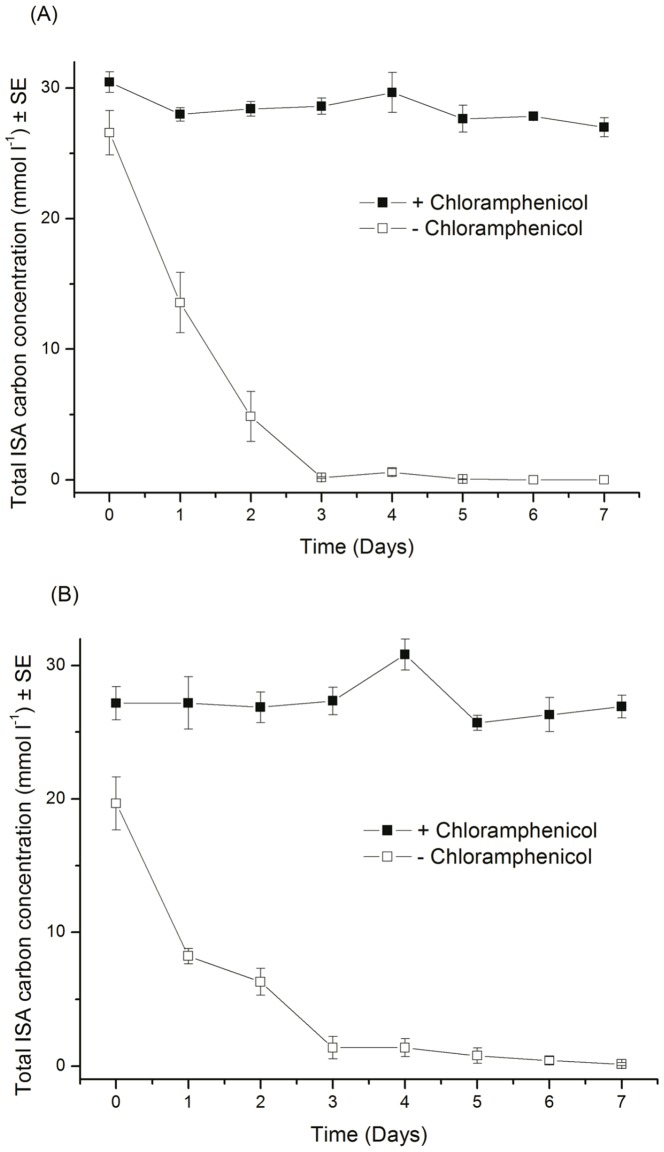
Fate of ISA's in microcosms treated with chloramphenicol when compared with untreated microcosm (A) ISA in presence of canal sediment and (B) reed bed sediment (n = 3).

ISA is known to be subject to sorption and precipitation reactions [7], [20], consequently a set of control microcosms treated with 50 µg ml^−1^ chloramphenicol were sampled over the same period and analysed for ISA content. In this instance, ISA was not removed over the seven day sample period (Figure 7), suggesting that the removal previously seen was microbially mediated rather than through sorption or precipitation processes. Other organic carbon sources were present within the CDP feed stock (<30% of total carbon) including the xylo-isosaccharinic acid and the α and β metasaccharinic acids. These minor components are degraded in all three systems (data not shown), however the CDP did contain recalcitrant components that remained un-degraded throughout the incubation period.

## Conclusions

Although the α and β forms of ISA are not naturally observed in the wider environment, bacteria found in anoxic sediments are capable of degrading these compounds by utilising a range of terminal electron acceptors at circa neutral pH. Under iron reducing, sulphate reducing and methanogenic conditions the degradation of ISA followed the pathway seen in anoxic environments driven by the degradation of polymeric organic materials; i.e. the fermentation of polymer monomers followed by the degradation of fermentation end products by terminal electron accepting processes. In this case, however, hydrolysis is a chemical rather than a microbial process. The persistence of bacteria commonly associated with the anaerobic degradation of cellulose (the Clostridia) in these batch fed microcosms suggests that they may play an important role in the metabolism of ISA into common fermentation end products allowing electron and carbon flow within these systems. In summary, these findings indicate that the ability to degrade ISA is common in near-surface microbial communities and consequently such communities represent a potential source of ISA degrading consortia for the colonisation of a GDF during the operational and pre-closure period.

The observed rates of ISA degradation suggest that at the interface between neutral and alkaline environments (e.g. within ungrouted wastes) ISA production will be the rate limiting step and that microbial activity will prevent the accumulation and transport of ISA and therefore prevent the enhanced migration of radionuclides. However, the activity of these communities within a GDF will be dependent on either the establishment of low pH environments within ungrouted wastes and/or their ability to adapt to the prevailing alkaline conditions. Consequently, ISA may persist, migrate and complex in regions where the pH inhibits microbial activity.

## Supporting Information

File S1 **Combined file containing supporting figures and tables.** Figure S1: XRD pattern from iron (III) oxide used in this study. Overlaid red lines indicate the allowed positions of the Bragg peaks for hematite, from the Powder Diffraction file database (Joint Committee of Powder Diffraction, JCPDS card number 89–0599. Figure S2: Non acetic volatile fatty acid concentrations in (A) iron reducing reactors, (B) sulphate reducing reactors and (C) methanogenic reactors. Figure S3: EDS output from analysis of calcite deposit in Figure 3, D. Table S1: Composition of cellulose degradation products. Table S2: PCR primers used in this study. Table S3: Organisms used as positive controls for PCR studies.
(DOCX)
