# Supplementary material for: Biodegradation of the Alkaline Cellulose Degradation Products Generated during Radioactive Waste Disposal
Source: PLoS One. 2014 Sep 30;9(9):e107433. doi: 10.1371/journal.pone.0107433 (PMC4182033; doi:10.1371/journal.pone.0107433)
Supplement: File S1 — Combined file containing supporting figures and tables. Figure S1: XRD pattern from iron (III) oxide used in this study. Overlaid red lines indicate the allowed positions of the Bragg peaks for hematite, from the Powder Diffraction file database (Joint Committee of Powder Diffraction, JCPDS card number 89–0599. Figure S2: Non acetic volatile fatty acid concentrations in (A) iron reducing reactors, (B) sulphate reducing reactors and (C) methanogenic reactors. Figure S3: EDS output from analysis of calcite deposit in Figure 3, D. Table S1: Composition of cellulose degradation products. Table S2: PCR primers used in this study. Table S3: Organisms used as positive controls for PCR studies. (DOCX) [file pone.0107433.s001.docx]

Supporting information


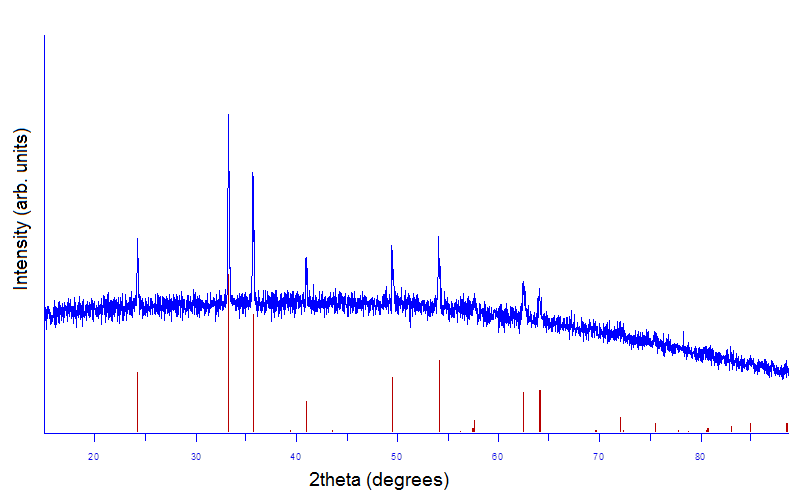


Figure S1: XRD pattern from iron (III) oxide used in this study. Overlaid red lines indicate the allowed positions of the Bragg peaks for hematite, from the Powder Diffraction file database (Joint Committee of Powder Diffraction, JCPDS card number 89-0599)


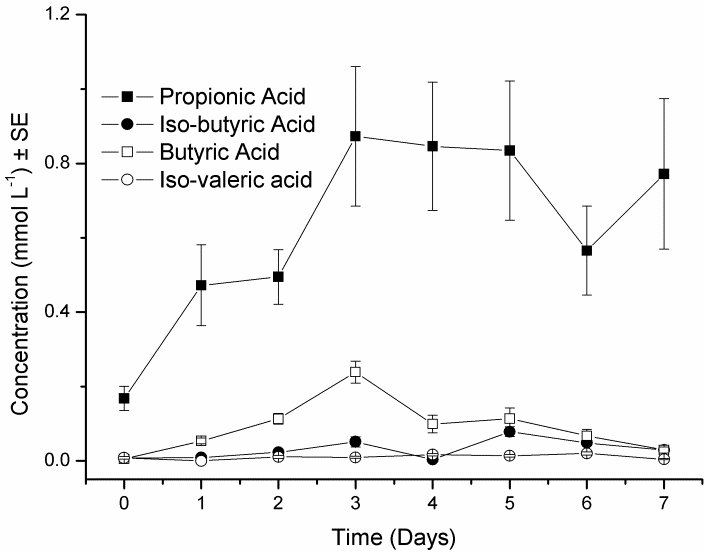

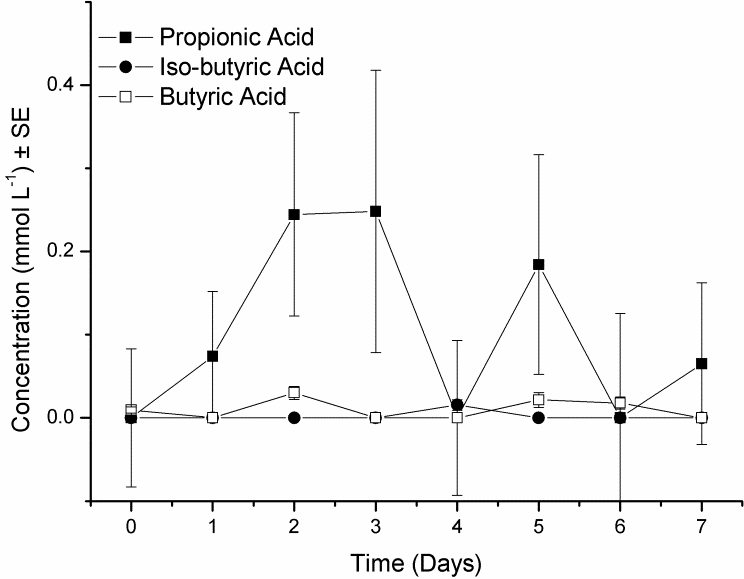

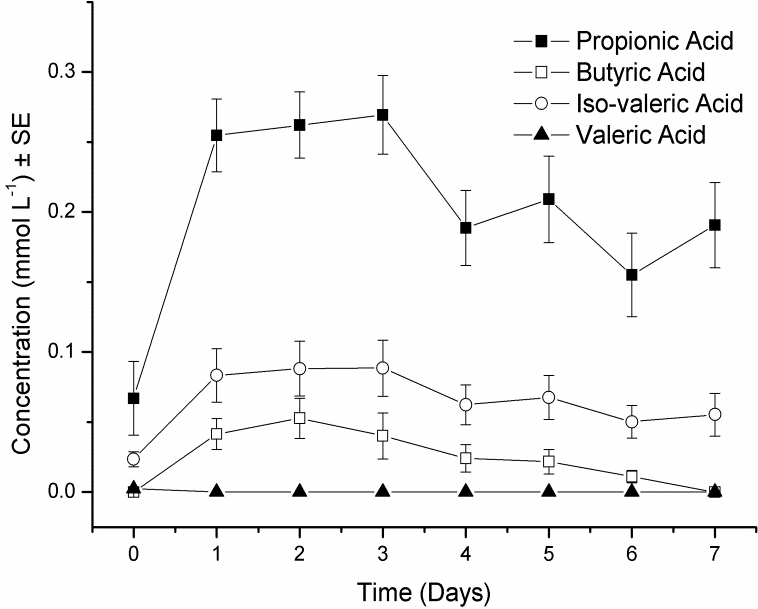


A

B

C

Figure S2 Non acetic volatile fatty acid concentrations in (A) iron reducing reactors, (B) sulphate reducing reactors and (C) methanogenic reactors.


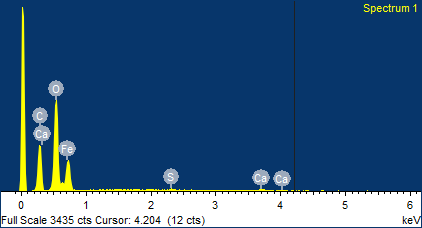


Internal Standard

Figure S3: EDS output from analysis of calcite deposit in Figure 3, D

| **Component** | **% of Total organic carbon** |
| --- | --- |
|  |  |
| α-Isosaccharinic acid | 40.00 |
| β-Isosaccharinic acid | 34.29 |
| Xylo-isosaccharinic acid | 3.37 |
| α and β Metasaccharinic acids | 3.23 |
| Formic Acid | 0.57 |
| Acetic acid | 0.29 |
| Propionic acid | 0.03 |
| Isovaleric acid | 0.06 |
| Butyric acid | 0.01 |
| Hydroxybutyric acid | * |
| Octanedioic acid derivative | * |
| Total | 81.84 |
|  |  |
| *Detected by MS but not quantified | |

Table S1: Composition of cellulose degradation products.

| Target Group | Primer set | Sequence (5'-3') | Size (bp) | Reference |
| --- | --- | --- | --- | --- |
|  |  |  |  |  |
| Eubacterial 16S rDNA | PA | AGAGTTTGATCCTGGCTAG | 1534 | [1] |
|  | PH' | AAGGAGGTGATCCAGCCGCA |  |  |
| Archeael 16S rDNA | Af | CCCTAYGGGGYGCASGAG | 660 | [2] |
|  | Ar | GGGCATGCACYWCYTCTC |  |  |
| Clostridium cluster I | Chis 150 | TTATGCGGTATTAATCTYCCTTT | 820 | [3] |
|  | Chot 983 | CARGRGATGTCAAGYCYAGGT |  |  |
| Clostridium cluster III | Cther 650 | TCTTGAGTGYYGGAGAGGAAAGC | 720 | [3] |
|  | Cther 1352 | GRCAGTATDCTGACCTRCC |  |  |
| Clostridium cluster IV | Clos 561 | TTACTGGGTGTAAAGGG | 580 | [3] |
|  | Clept 1129 | TAGAGTGCTCTTGCGTA |  |  |
| Clostridium cluster XIVab | Erec 482 | GCTTCTTAGTCARGTACC | 620 | [3] |
|  | Cooc 112 | TGGCTACTRFRVAYARG |  |  |
| Methanococcales | MCC495F | TAAGGGCTGGGCAAGT | 340 | [4] |
|  | MCC832R | CACCTAGTYCGCARAGTTTA |  |  |
| Methanobacteriales | MBT857F | CGWAGGGAAGCTGTTAAGT | 345 | [4] |
|  | MBT1196R | TACCGTCGTCCACTCCTT |  |  |
| Methanomicrobiales | MMB282f | ATCGR TACGGGTTGTGGG | 506 | [4] |
|  | MMB832R | CACCTAACGCRCATHGTTAC |  |  |
| Methanosarcinales | MSL812F | GTAAACGATRYTCGCTAGGT | 350 | [4] |
|  | MSL1159R | GGTCCCCACAGWGTACC |  |  |
| Methanosaeta | MS1585F | CCGGCCGGATAAGTCTCTTGA | 270 | [5] |
|  | SAE835R | GACAACGGTCGCACCGTGGCC |  |  |
| SRB group 1 | DFM140 | TAGMCYGGGATAACRSKYG | 702 | [6] |
|  | DFM842 | ATACCCSCWWCWCCTAGCAC |  |  |
| SRB group 2 | DBB121 | CGCGTAGATAACCTGTCYTCATG | 1120 | [6] |
|  | DBB1237 | GTAGKACGTGTGTAGCCCTGGTC |  |  |
| SRB group 3 | DBM169 | CTAATRCCGGATRAAGTCAG | 840 | [6] |
|  | DBM1006 | ATTCTCARGATGTCAAGTCTG |  |  |
| SRB group 4 | DSB127 | GATAATCTGCCTTCAAGCCTGG | 1150 | [6] |
|  | DSB1273 | CYYYYYGCRRAGTCGSTGCCCT |  |  |
| SRB group 5 | DCC305 | GATCAGCCACACTGGRACTGACA | 860 | [6] |
|  | DCC1165 | GGGGCAGTATCTTYAGAGTYC |  |  |
| SRB group 6 | DSV230 | GRGYCYGCGTYYCATTAGC | 610 | [6] |
|  | DSV838 | SYCCGRCAYCTAGYRTYCATC |  |  |
| *G. metallireducens* | GEOF | ATGGCGAGAACAGACGAG | 300 | [7] |
|  | GEOR | CTTCTGCGCCGTCGGC |  |  |
| *Shewanella* sp | She211f | CGCGATTGGATGAACCTAG | 1040 | [8] |
|  | She1259r | GGCTTTGCAACCCTCTGTA |  |  |

Ambiguities: R (G or A); Y (C or T); K (G or T); M (A or C); S (G or C); W (A or T).

Table S2: PCR primers used in this study

| Target Group | Control DNA |
| --- | --- |
|  |  |
| Eubacterial 16S rDNA | *Clostridium pasteurianum* DSM525 |
|  |  |
| Archeael 16S rDNA | *Methanococcus voltae* DSM1537 |
|  |  |
| Clostridium cluster I | *Clostridium pasteurianum* DSM525 |
|  |  |
| Clostridium cluster III | *Clostridium termitidis* DSM5398 |
|  |  |
| Clostridium cluster IV | *Clostridium sporosphaeroides* DSM1294 |
|  |  |
| Clostridium cluster XIVab | *Clostridium celerecrescens* DSM5628 |
|  |  |
| Methanococcales | *Methanococcus voltae* DSM1537 |
|  |  |
| Methanobacteriales | *Methanobacterium bryantii* DSM863 |
|  |  |
| Methanomicrobiales | *Methanomicrobium mobile* DSM1539 |
|  |  |
| Methanosarcinales | *Methanosarcina acetivorans* DSM2834 |
|  |  |
| Methanosaeta | *Methanosaeta pelagica* DSM24271 |
|  |  |
| SRB group 1 | *Desulfobulbus propionicus* DSM2032 |
|  |  |
| SRB group 2 | *Desulfobacterium niacini* DSM2650 |
|  |  |
| SRB group 3 | *Desulfobacter postgatei* DSM2034 |
|  |  |
| SRB group 4 | *Desulfococcus multivorans* DSM2059 |
|  |  |
| SRB group 5 | *Desulfovibrio africanus* DSM2603 |
|  |  |
| SRB group 6 | *Desulfotomaculum nigrificans* DSM574 |
|  |  |
| *G. metallireducens* | *Geobacter metallireducens* DSM7210 |
|  |  |
| *Shewanella* sp | *Shewanella putrefaciens* DSM6067 |
|  |  |

Table S3: Organisms used as positive controls for PCR studies

References

1. Edwards U, Rogall T, Blöcker H, Emde M, Böttger EC (1989) Isolation and direct complete nucleotide determination of entire genes. Characterization of a gene coding for 16S ribosomal RNA. *Nucleic Acids Research* 17: 7843-7853.

2. Gantner S, Andersson AF, Alonso-Sáez L, Bertilsson S (2011) Novel primers for 16S rRNA-based archaeal community analyses in environmental samples. *Journal of Microbiological Methods* 84: 12-18.

3. Van Dyke MI, McCarthy AJ (2002) Molecular Biological Detection and Characterization of Clostridium Populations in Municipal Landfill Sites. *Applied and Environmental Microbiology* 68: 2049-2053.

4. Yu Y, Lee C, Kim J, Hwang S (2005) Group-specific primer and probe sets to detect methanogenic communities using quantitative real-time polymerase chain reaction. *Biotechnology and Bioengineering* 89: 670-679.

5. Shigematsu T, Tang Y, Kawaguchi H, Ninomiya K, Kijima J, et al. (2003) Effect of dilution rate on structure of a mesophilic acetate-degrading methanogenic community during continuous cultivation. *Journal of Bioscience and Bioengineering* 96: 547-558.

6. Daly K, Sharp RJ, McCarthy AJ (2000) Development of oligonucleotide probes and PCR primers for detecting phylogenetic subgroups of sulfate-reducing bacteria. *Microbiology* 146: 1693-1705.

7. Kuntze K, Shinoda Y, Moutakki H, McInerney MJ, Vogt C, et al. (2008) 6-Oxocyclohex-1-ene-1-carbonyl-coenzyme A hydrolases from obligately anaerobic bacteria: characterization and identification of its gene as a functional marker for aromatic compounds degrading anaerobes. *Environmental Microbiology* 10: 1547-1556.

8. Todorova SG, Costello AM (2006) Design of Shewanella-specific 16S rRNA primers and application to analysis of Shewanella in a minerotrophic wetland. *Environmental Microbiology* 8: 426-432.
